# Small-Molecule Inhibitors Targeting Bacterial Antioxidants Potentiate Bactericidal Efficacy of Cold Atmospheric Plasma Against *Staphylococcus aureus*

**DOI:** 10.3390/antiox15101316

**Published:** 2026-10-12

**Authors:** Ruolan Ding, Li Tan, Xitong Zuo, Xiaonan Huang, Qishen Liang, Zhen Hu, Yanqiao Li, Weilong Shang, Wanzhen Xiao, Huagang Peng, Ziqi Zhang, Yuting Wang, Zexian Xu, Xiancai Rao, Yi Yang

**Affiliations:** 1 Department of Microbiology, College of Basic Medical Sciences, Key Laboratory of Microbial Engineering Under the Educational Committee in Chongqing, Army Medical University, Chongqing 400038, China; 30027209@alu.cqu.edu.cn (R.D.); tanli@tmmu.edu.cn (L.T.); 18301933539@163.com (Q.L.); huzhenzhen1314@tmmu.edu.cn (Z.H.); shangwl@tmmu.edu.cn (W.S.); huagangpeng@tmmu.edu.cn (H.P.); wangyuting@tmmu.edu.cn (Y.W.); 2 College of Basic Medical Sciences, Army Medical University, Chongqing 400038, China; 15133302736@163.com (X.Z.); yanqiaoli666@tmmu.edu.cn (Y.L.); manyinshi@tmmu.edu.cn (W.X.); ziqizhang@tmmu.edu.cn (Z.Z.); xzxyx@tmmu.edu.cn (Z.X.); 3 State Key Laboratory of Pathogen and Biosecurity, Beijing Institute of Microbiology and Epidemiology, Beijing 100071, China; huangxn1999@126.com

**Keywords:** *Staphylococcus aureus*, cold atmospheric plasma, anti-infection therapy, reactive oxygen/nitrogen species, small molecule inhibitor

## Abstract

Antibiotic-resistant *Staphylococcus aureus* infections pose a formidable clinical challenge. Cold atmospheric plasma (CAP) has emerged as a promising disinfection technology; however, the underlying bacterial response mechanisms and potential resistance to CAP remain incompletely elucidated. Here, we validated the broad-spectrum and efficient bactericidal activity of CAP against both drug-sensitive and drug-resistant clinical isolates of *S. aureus*. Mechanistically, CAP primarily eliminated bacteria by inducing oxidative damage to proteins and lipids, thereby disrupting internal cellular structures. The addition of exogenous free radical scavengers significantly attenuated CAP-mediated oxidative killing. Transcriptomic analysis and RT-qPCR validation further revealed upregulation of redox-related genes in *S. aureus* following CAP exposure. Deletion of key antioxidant genes, particularly *ahpC* and *mrgA*, markedly enhanced bacterial susceptibility to CAP. Virtual screening and surface plasmon resonance analysis identified two small-molecular inhibitors, C4515 and A8226, which specifically target AhpC and MrgA, respectively, and potentiated CAP bactericidal efficacy. Collectively, this study delineates the molecular interactions between CAP and *S. aureus*, and underscores the potential of CAP-based strategies for combating drug-resistant bacterial infections.

## 1. Introduction

Bacterial infections impose a substantial burden on global public health and socio-economic development [1,2]. Despite the widespread availability of antibiotics, the emergence and global spread of antibiotic-resistant microbes (ARMs), particularly ESKAPE pathogens and other superbugs, have drastically challenged infection control [3]. According to The Lancet, 13.7 million infection-related deaths in 2019 were attributed to 33 major clinical bacterial pathogens, with nearly 36% (approximately 4.95 million) linked to ARMs [4,5]. Alarmingly, the global burden of antimicrobial resistance is projected to escalate, with cumulative deaths from ARM infections expected to reach 92.0 million over the next 25 years [6]. Given this trajectory and the persistent “arms race” between ARMs and antibiotic development, exploring alternative therapeutic strategies beyond conventional antibiotics is urgently needed [7].

Cold atmospheric plasma (CAP) represents an emerging bactericidal technology [8,9]. It generates diverse active species, including ultraviolet photons, charged particles, reactive oxygen species (ROS), and reactive nitrogen species (RNS) [10], and exerts broad-spectrum sterilization effects against clinically relevant bacteria [11]. CAP has been widely applied in food decontamination, environmental disinfection, and clinical anti-infective therapy [12,13,14]. However, as a nascent anti-infective approach, CAP requires further exploration and refinement for in vivo application. CAP kills bacteria primarily by inducing oxidative damage [15,16], yet bacterial sensitivity to CAP varies significantly [17,18,19], with some isolates showing reduced susceptibility to low-intensity CAP treatment. Consequently, higher CAP intensities are often required to achieve complete disinfection, which raises safety concerns about potential oxidative damage to human tissues. Attenuating bacterial resistance to CAP is therefore pivotal for balancing bactericidal efficacy and safety. Notably, studies on bacterial defense mechanisms against CAP remain limited, highlighting the significance of identifying key bacterial effectors that mediate CAP–bacteria interactions and leveraging these factors to enhance bacterial susceptibility to CAP.

In this study, we used *Staphylococcus aureus*, a clinically prominent drug-resistant bacterium [20], as a model to evaluate CAP-mediated sterilization. CAP demonstrated comparably potent disinfection effects against both drug-resistant and drug-sensitive clinical *S. aureus* isolates, acting mainly through a ROS/RNS-dependent mechanism that damages internal bacterial components without disrupting external structures. In response, *S. aureus* counteracts CAP-mediated killing by upregulating antioxidant factors, with AhpC and MrgA playing pivotal roles. Using virtual screening and surface plasmon resonance (SPR), we identified C4515 and A8226 as candidate small-molecule inhibitors that specifically block the enzymatic activities of AhpC and MrgA, respectively. Notably, combining these inhibitors with CAP significantly enhanced in vitro bactericidal efficacy against *S. aureus*. Overall, our findings support the CAP-small molecule combination as a promising strategy for combating ARM infections, warranting further research to explore its clinical potential.

## 2. Material and Methods

### 2.1. Bacterial Strains

Seventy-two clinical *S. aureus* isolates (designated TJ001–TJ072) were provided by Dr. Dongmei Wang (983th hospital of PLA) [21], and cultivated in brain heart infusion (BHI) broth with shaking at 37 °C. Other *S. aureus* strains, including Newman, RN4220, USA300, and XN108 (Table S1), were cultivated in tryptic soy broth (TSB) unless otherwise specified. *Escherichia coli* strains DH5α and BL21(DE3) were purchased from Beijing Biomed Gene technology Co., Ltd. (Beijing, China). and cultured in Luria–Bertani (LB) medium.

### 2.2. CAP Device

A dielectric barrier discharge (DBD)-type CAP device, designed for test-tube-based treatments, was used as previously described [22]. The operating parameters were set as follows: constant output power of 20 W (25 V, 0.8 A), treatment duration ranging from 0–5 min, and a fixed distance of 0.5 cm between the bacterial sample and the electrode tip. All CAP treatments were performed in an air-conditioned room with a relative humidity of 40% ± 1% at room temperature.

### 2.3. CAP Treatment Assay

Clinical *S. aureus* isolates were grown in BHI, whereas laboratory-passaged strains were cultured in TSB. Overnight bacterial cultures were diluted 1:100 into their respective medium and incubated for an additional 6 h. Bacterial cells were harvested by centrifugation. After discarding the supernatant, bacterial pellets were washed twice with sterile normal saline (NS), resuspended in NS, and adjusted to a turbidity of 0.5 McFarland standard (approximately 1.0 × 10^8^ CFU/mL). Equal volumes of bacterial suspension were exposed to CAP for 0, 1, 3, or 5 min, then incubated at room temperature for 1 h. Viable bacterial counts were determined using the plate dilution method [23]. The relative survival index (RSI) = log_10_(survival count after CAP)/log_10_(initial count before CAP).

For treatment of purified biomolecules: Bacterial genomic DNA was extracted using the TIANamp Bacteria DNA Kit (Tiangen, Beijing, China). Proteins were prepared by harvesting overnight cultures, resuspending cells in ice cold NS containing 1% (*w*/*v*) β-mercaptoethanol (Sigma-Aldrich, St. Louis, MO, USA) and 1 mM phenylmethylsulfonyl fluoride (PMSF) (Beyotime, Shanghai, China), and lysing with 0.1 mm diameter zirconia–silica beads in a Minibeadbeater 16 (Biospec, Bartlesville, OK, USA). Supernatants were collected by centrifugation. Equal amounts of DNA or protein were treated with CAP for 0, 1, 3, or 5 min. DNA integrity was analyzed by 1% agarose gel electrophoresis and quantified with a NanoDrop One (Thermo Fisher Scientific, Waltham, MA, USA). Protein integrity was assessed by SDS-PAGE and quantified with a BCA Protein Assay kit (Beyotime, Shanghai, China).

### 2.4. Bacterial Growth Curve

Overnight *S. aureus* cultures (with or without prior CAP treatment) were diluted 1:100 in 100 mL of fresh TSB and incubated at 37 °C with shaking. The OD_600_ was measured hourly using a Multiskan GO spectrophotometer (Thermo Fisher Scientific, Waltham, MA, USA). Growth curves were plotted using GraphPad Prism 8.0.1.

### 2.5. SEM Observation

Bacterial surface integrity after CAP treatment was evaluated by SEM as described [23]. Briefly, CAP-treated cells were dropped onto clean coverslips, fixed with 2.5% (*v*/*v*) glutaraldehyde at 4 °C overnight, washed twice with NS, and dehydrated in graded ethanol solutions (30%, 50%, 60%, 70%, 90%; 5 min each) and rinsed three times in 100% ethanol (7 min each). Ethanol was replaced with graded tert-butyl alcohol (30%, 50%, 70%, 90%, 100%). Air-dried samples were sputter-coated with gold and examined using a Hitachi S-3400N II SEM (Hitachi, Tokyo, Japan).

### 2.6. TEM Observation

Bacterial internal structural changes were evaluated by TEM. Briefly, bacterial cells (with or without CAP) were pelleted, fixed with 2.5% glutaraldehyde at 4 °C overnight, washed three times with PBS (15 min each), post-fixed with 1% OsO_4_ for 2 h, washed again, and dehydrated through an ethanol series (50%, 70%, 90%, 100%; 5 min each), followed by two acetone rinses (10 min each). Samples were embedded in resin at 25 °C for 4 h, followed by polymerization at 65 °C for 48 h. Ultrathin sections were prepared using an EM UC7 ultramicrotome (Leica, Wetzlar, Germany), stained with uranyl acetate for 20 min and alkaline lead citrate for 10 min, and observed under a TECNAI 10 TEM (Philips, Eindhoven, The Netherlands).

### 2.7. Detection of Reactive Species and Bacterial Oxidative Damage

ROS and RNS levels in CAP-activated systems were quantified after 0, 1, 3, and 5 min exposure.

Hydrogen peroxide (H_2_O_2_) was quantified using the Amplex^®^ Red Hydrogen Peroxide/Peroxidase Assay Kit (Invitrogen, Carlsbad, CA, USA). Amplex Red reagent reacts with H_2_O_2_ in the presence of horseradish peroxidase (HRP) to form resorufin (excitation/emission maxima: ~571 nm/~585 nm). The OD_590_ value of the reaction mixture was detected using a Multiskan GO spectrophotometer (Thermo Fisher Scientific, Waltham, MA, USA).

Nitric oxide (NO) was detected using a Nitric Oxide assay kit (njjcbio, Nanjing, China). NO is rapidly converted to NO_2_^−^ and NO_3_^−^, with NO_2_^−^ further converted to NO_3_^−^. Nitrate reductase was utilized to reduce NO_3_^−^ to NO_2_^−^, and the OD_550_ value was measured.

Hydroxyl radical (•OH) was assessed using phthalic acid (PA; Sigma-Aldrich, St. Louis, MO, USA). NS supplemented with PA was treated with CAP for different durations. •OH reacts with PA to form 2-hydroxyterephthalic acid (HTA), a stable fluorescent compound. OD_455_ was recorded.

Lipid oxidative damage was assessed by measuring malondialdehyde (MDA; Lipid Peroxidation MDA Assay Kit, Beyotime, Shanghai, China), and protein oxidative damage by protein carbonylation (Protein Carbonyl Detection Kit, PUMOKE, Wuhan, China), following the manufacturer’s instructions. All experiments were independently repeated three times.

### 2.8. ROS Scavenging Assays

The role of reactive species in CAP killing was evaluated using scavengers: reduced L-glutathione (L-GSH), hemoglobin (HEMO), sodium pyruvate (SP), and mannitol (Man), all from Sigma-Aldrich (St. Louis, MO, USA). For L-GSH: *S. aureus* Newman suspension (1 × 10^8^ CFU/mL) was aliquoted (500 μL/tube). Tube 1: no additive, no CAP (negative control). Tubes 2–5: 0, 1, 10, and 100 mM L-GSH + 5 min CAP. Tube 6: 100 mM L-GSH only (antioxidant control). After 1 h incubation at room temperature, viable counts were determined using the plate dilution method.

The effects of HEMO (0, 0.01, 0.1, 1 mM), SP (0, 1, 10, 100 mM), and Man (0, 0.01, 0.1, 1 mM) on CAP-mediated *S. aureus* killing were assessed similarly. All experiments were repeated three times.

### 2.9. RNA Sequencing

Overnight *S. aureus* Newman culture was diluted 1:100 in fresh TSB and cultured at 37 °C for 6 h. Bacterial cells were harvested by centrifugation, resuspended in sterile NS, and divided into six aliquots (three CAP-treated for 5 min, three untreated controls). Total RNA was extracted using the RNAprep Pure Cell/Bacteria Kit (TianGen, Beijing, China). Libraries were constructed and sequenced on the Illumina Hiseq Novaseq-PE150 platform (Novogene Bioinformatics Technology Co., Ltd., Beijing, China). Reads were mapped to the *S. aureus* Newman genome (GenBank accession: AP009351.1). Differentially expressed genes (DEGs) were identified using the DESeq2 package with thresholds of |log2FoldChange| > 0.0 and padj < 0.05. Kyoto Encyclopedia of Genes and Genomes (KEGG) pathway enrichment analysis was performed using ClusterProfiler (v4.6.0) software with Fisher’s exact test, and false discovery rate (FDR) correction for multiple testing. KEGG pathways with *p* < 0.05 were considered statistically significant.

The raw data were submitted to the sequence read archive (SRA) database with an accession number of PRJNA1503662.

### 2.10. RT-qPCR

Expression of antioxidant genes was quantified by real-time quantitative reverse transcription polymerase chain reaction (RT-qPCR). In brief, bacterial samples were prepared as described above and treated with CAP for 5 min. Untreated cells served as negative controls. Total RNA was extracted using the SV Total RNA Isolation System (Promega, Madison, WI, USA). Genomic DNA was removed using RQ1 RNase-free DNase (Promega, Madison, WI, USA), and cDNA was synthesized using the RevertAid First Strand cDNA Synthesis Kit (Thermo Fisher Scientific, Waltham, MA, USA). Target gene expression was detected with 16S rRNA as the reference gene for normalization. Relative gene expression in controls was set to 1.0. All primers are listed in Table S2.

### 2.11. Construction of Gene Deletion Mutants

Mutants were constructed by homologous recombination. For Newman-Δ*sodA*: Approximately 1000 bp DNA fragments flanking *sodA* gene were amplified with primer pairs *sodA*-ko-1-1/-2 and *sodA*-ko-2-1/-2, cloned into the temperature-sensitive shuttle vector pBT2, electroporated into *S. aureus* RN4220 for restriction modification, then into *S. aureus* Newman. Mutants were selected by alternating culture at 42 °C (with 20 μg/mL chloramphenicol) and 25 °C. Correct deletions were verified by PCR using primer pair *sodA*-out-1/-2 and sequencing. Other mutants were constructed analogously; all primers are listed in Table S2.

### 2.12. Preparation of Recombinant Proteins

Recombinant AhpC protein was prepared using a prokaryotic expression system. Briefly, the *ahpC* coding sequence was amplified from *S. aureus* Newman genomic DNA using primer pair *ahpC*-cds-1/-2 (Table S2), and cloned into pET28a. The resulting plasmid pET28a-*ahpC* was transformed into *E. coli* BL21(DE3) and verified by DNA sequencing. AhpC expression was induced by 0.5 mM isopropylthio-β-D-galactoside (IPTG) (Sigma-Aldrich, St. Louis, MO, USA). The expressed protein was purified using a HisTrap HP column (Cytiva, Marlborough, MA, USA). Protein purity was checked by SDS-PAGE, and concentration was measured using an Enhanced BCA Protein Assay Kit (Beyotime, Shanghai, China). Recombinant MrgA was prepared similarly. Primers are listed in Table S2.

### 2.13. In Vitro Enzyme Activity and Inhibitor Assays

AhpC peroxidase activity was determined using a modified xylenol orange (FOX) assay [24]. Briefly, purified AhpC was pre-mixed with 1 mL of 0.05 mM PBS buffer (containing 1 mM dithiothreitol, DTT) in Eppendorf (EP) tubes. *t*-Butyl hydroperoxide (*t*-BHP) was added to a final concentration of 40 μM. Tubes containing AhpC alone or *t*-BHP in PBS served as controls. Every 5 min, 50-μL aliquots were mixed with 650 μL of FOX reagent (250 μM ammonium ferrous sulfate, 125 μM xylenol orange, 100 μM sorbitol, and 25 mM sulfuric acid) and incubated at room temperature for 30 min. Color changes were recorded, and the OD_560_ value was measured using a Multiskan GO spectrophotometer (Thermo Fisher Scientific, Waltham, MA, USA).

MrgA ferroxidase activity was measured as previously described [25]. Briefly, purified MrgA was mixed with reaction buffer (50 mM MOPS, 150 mM NaCl, pH 7.0) and degassed by argon bubbling to eliminate dissolved oxygen. Ferrous ammonium sulfate (Fe^2+^ source) was added, and the OD_310_ value was measured immediately using a Multiskan GO spectrophotometer (Thermo Fisher Scientific, Waltham, MA, USA). Bovine serum albumin (BSA; Sigma-Aldrich, St. Louis, MO, USA) was used as a negative protein control.

To evaluate the inhibitory effects of candidate small molecules on AhpC and MrgA activity: Different concentrations of each small molecule were pre-incubated with the target protein at 37 °C for 30 min, followed by enzyme activity determination using the protocols described above.

### 2.14. Virtual Screening

Small-molecule inhibitors targeting AhpC were identified via molecular docking-based virtual screening. The initial structure of AhpC was predicted using AlphaFold3 (UniProt ID: P0A0B7). Protein structure processing and virtual screening were performed using Autodock Vina. The commercial ligand library L-CO-020 (APExBIO, approximately 10,600 compounds) was used for screening; compounds were converted to three-dimensional (3D) structures using the Open Babel toolkit. A docking box was centered on the catalytic domain of AhpC to ensure reasonable spatial interaction between small molecules and the protein. Virtual screening was conducted as follows: All candidate compounds were docked to AhpC, and docking scores were calculated using the default parameters of AutoDock Vina (v1.2.0). Compounds were then ranked based on their docking scores.

The same strategy was employed to identify MrgA inhibitors, using the AlphaFold3-predicted-structure of MrgA (UniProt ID: A0A0H2X069).

### 2.15. SPR Binding Assay

Binding kinetics were measured on a Biacore T200 (Cytiva, Uppsala, Sweden) using a Series S Sensor Chip CM5 (Cytiva, Uppsala, Sweden). The running buffer was PBST. Ligand immobilization was performed by activating the CM5 chip with a 1:1 mixture of 0.4 M EDC and 0.1 M NHS (Amine Coupling Kit, Cytiva, Uppsala, Sweden) at a flow rate of 10 μL/min. AhpC was injected in 10 mM sodium acetate (pH 4.0) until ~5000 resonance units (RU) were achieved. The chip surface was then blocked with ethanolamine at 10 μL/min. Small-molecule analytes were dissolved in PBST and serially diluted to concentrations ranging from 50 to 0.195 μM. Binding kinetics were measured at 25 °C using multi-cycle kinetics (flow rate: 30 μL/min; association time: 90 s; dissociation time: 300 s). Three startup cycles and blank injections were performed prior to sample analysis. Sensorgrams were double-referenced; kinetic parameters (*kon*, *koff*) and equilibrium dissociation constants (*K_D_*) were calculated using Biacore Evaluation Software (v3.2.1, Cytiva, Uppsala, Sweden) with global fitting to a 1:1 Langmuir binding model.

### 2.16. Combined Disinfection Assay

Candidate small-molecule inhibitors were combined with CAP to enhance disinfection efficacy. Briefly, bacterial samples were prepared as previously described, and adjusted with sterile NS to a final concentration of approximately 1.0 × 10^8^ CFU/mL. Aliquots (500 μL) were pre-incubated with inhibitors at the indicated concentrations for 30 min at 37 °C. The inhibitors used were A3237 (betulinic acid), B5866 (SCH772984 HCl), and C4515 (chlorothricin) at 5, 50, and 500 μM, and A8226 (TAK-632) at 0, 30, and 300 μM. Then, samples were CAP-treated for 90 s. After 1 h incubation, viable counts were determined by plate dilution.

For H_2_O_2_ combination, 200 μL aliquots were pre-incubated with inhibitors, treated with H_2_O_2_ for 30 min, and plated.

### 2.17. Statistics Analysis

Data analysis was performed using GraphPad Prism 8.0.1. Unpaired Student’s *t*-test was applied for comparisons between two independent groups. One-way analysis of variance (ANOVA) was employed for comparisons among three or more groups. Data are presented as mean ± standard deviation (SD). Statistical significance was defined as *p* < 0.05.

## 3. Results

### 3.1. CAP Exhibits Comparable Disinfection Efficacy Against Drug-Resistant and Drug-Susceptible S. aureus Isolates

**Figure 1:**
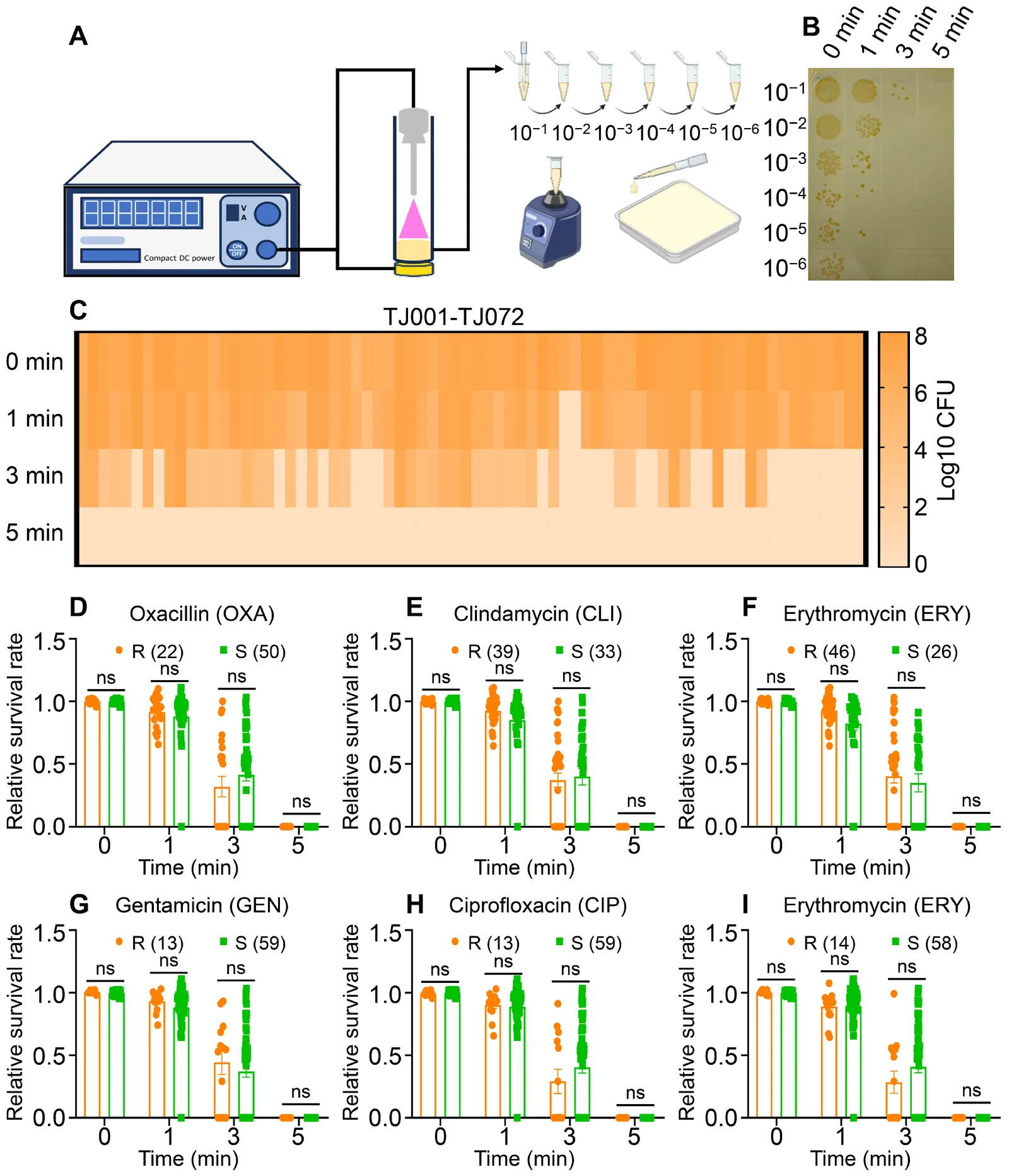
CAP exhibits effective bactericidal activity against clinical *S. aureus* isolates. (**A**) Workflow of the CAP treatment experiment, CAP-treated bacteria were 10-fold serially diluted and enumerated via a plate dilution method. (**B**) Representative image of bacterial counting via plate dilution method. (**C**) Heatmap showing the time-dependent bactericidal effects of CAP on 72 *S. aureus* isolates (TJ001–TJ072). (**D**–**I**) Comparison of CAP disinfection efficacy against antibiotic-resistant (R) and -sensitive (S) *S. aureus* strains grouped by resistance to: (**D**) oxacillin (OXA), (**E**) gentamicin (GEN), (**F**) clindamycin (CLI), (**G**) ciprofloxacin (CIP), (**H**) erythromycin (ERY), and (**I**) trimethoprim/sulfamethoxazole (SXT). Numbers in parentheses indicate the count of strains in each group. Data are expressed as mean ± standard deviation (SD). Statistical significance was calculated by Student’s *t*-test. “ns” indicates no significance.

CAP is known to effectively inactivate pathogenic bacteria [11]. In this study, 72 clinical *S. aureus* isolates were exposed to CAP, and bacterial survival was quantified via plate counting (Figure 1A). As shown in Figure 1B,C, CAP exhibited a time-dependent bactericidal effect. At an initial inoculum of ~1 × 10^8^ CFU/mL, viable bacterial counts remained nearly unchanged after 1 min of CAP exposure, apart from two strains that were fully inactivated. After 3 min exposure, 27 additional strains were fully eradicated, while the remaining 43 strains showed survival counts ranging from 3 to 7.15 log_10_ CFU/mL. A 5 min treatment achieved complete sterilization of all strains. These results confirm the rapid and efficient bactericidal activity of CAP against *S. aureus* and also reveal notable heterogeneity in CAP sensitivity across strains.

To determine whether the heterogeneity in CAP sensitivity correlates with antibiotic resistance phenotypes, we compared CAP efficacy against antibiotic-resistant (R) and -susceptible (S) isolates. According to oxacillin (OXA) susceptibility, the 72 strains were categorized into 22 R and 50 S isolates. Under identical conditions, the survival rates of R and S strains were nearly equivalent (Figure 1D). Consistent results were observed when strains were grouped by resistance to gentamicin (GEN), clindamycin (CLI), ciprofloxacin (CIP), erythromycin (ERY), or trimethoprim/sulfamethoxazole (STX) (Figure 1E–I). These findings indicate that bacterial sensitivity to CAP is independent of antibiotic resistance profiles, with CAP exhibiting comparable killing effects against both antibiotic-resistant and -susceptible *S. aureus* isolates.

### 3.2. CAP Induces Intracellular Cavitation and Protein Degradation in S. aureus

**Figure 2:**
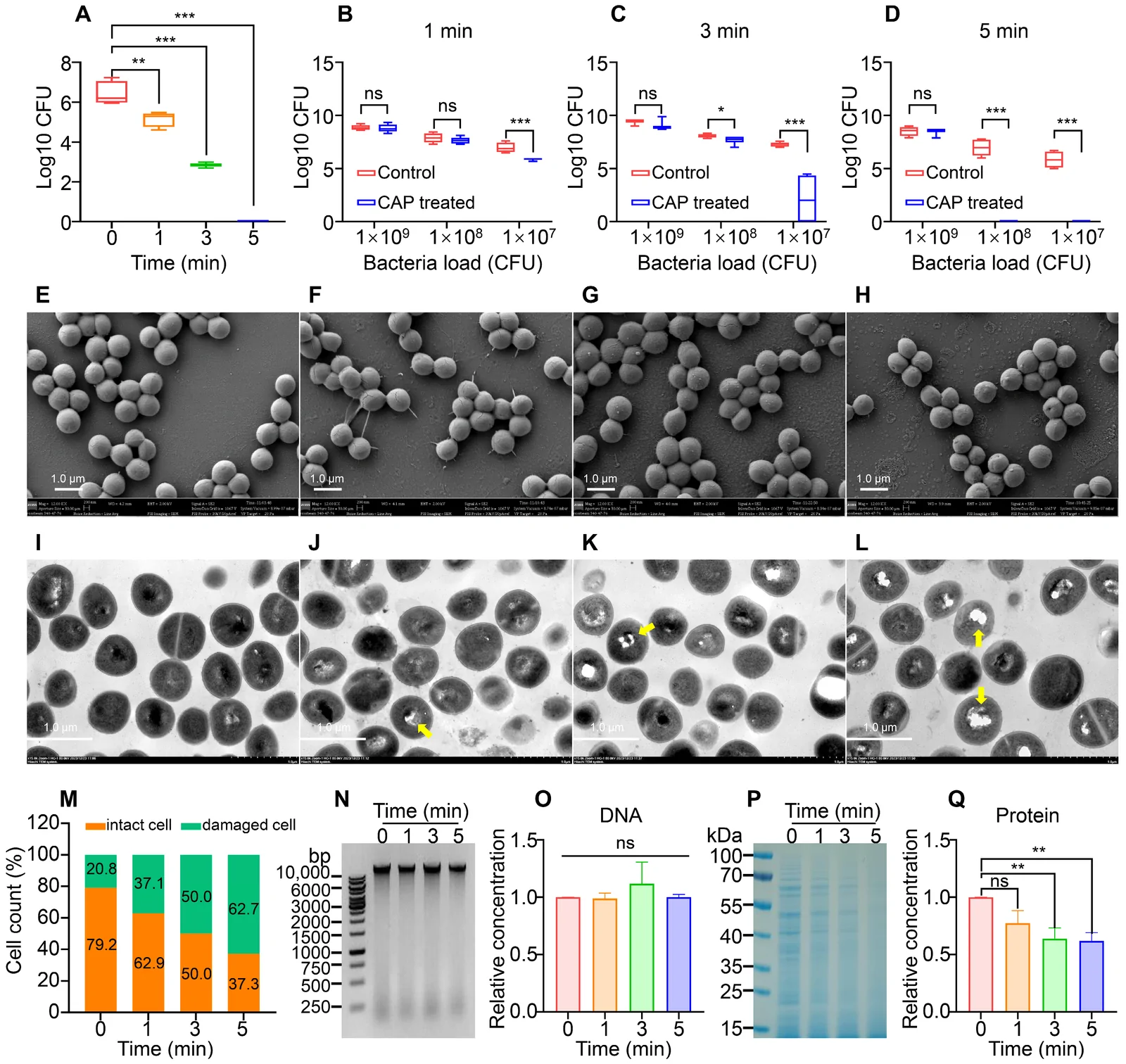
CAP induces intracellular damage and protein degradation in *S. aureus*. (**A**) Time-dependent bactericidal effect of CAP on *S. aureus* Newman. (**B**–**D**) Impact of initial bacterial load on CAP bactericidal efficacy. (**E**–**H**) SEM micrographs showing bacterial surface morphology after CAP exposure. (**I**–**L**) TEM micrographs showing CAP-induced intracellular morphological changes, as indicated by yellow arrows. (**M**) Quantification of intact vs. damaged cells from TEM images under different CAP conditions. (**N**) Analysis of DNA integrity by 1% agarose gel electrophoresis after CAP treatment. (**O**) Relative DNA concentrations after CAP treatment. (**P**) SDS-PAGE analysis of protein integrity following CAP treatment. (**Q**) Relative protein concentrations after CAP treatment. Data are presented as mean ± SD. Statistical significance was calculated by one-way ANOVA. “ns” indicates no significance, * *p* < 0.05, ** *p* < 0.01, *** *p* < 0.001.

Newman is a clinical *S. aureus* isolate with well-characterized genetic background and established genetic manipulation tools [26]. Notably, previous study has demonstrated its relative resistance to CAP [27], making it an ideal model for investigating CAP tolerance mechanisms. CAP treatment reduced bacterial loads by 1.35 and 3.58 log_10_ CFU/mL after 1 min and 3 min of exposure, respectively, with complete disinfection achieved at 5 min (Figure 2A). Interestingly, only a 5 min CAP treatment abolished bacterial cultivability; 1 min and 3 min exposures allowed surviving bacteria to recover to control-level counts (Figure S1). Moreover, the initial bacterial density significantly affected CAP efficacy: bacteria at 1 × 10^9^ CFU/mL exhibited greater CAP resistance, while those at 1 × 10^7^ CFU/mL were most susceptible (Figure 2B–D).

To evaluate CAP-induced morphological changes, we employed scanning electron microscopy (SEM) and transmission electron microscopy (TEM). SEM revealed that CAP exposure, regardless of duration, had minimal impact on bacterial surface structures (Figure 2E–H). In contrast, TEM images showed prominent intracellular cavitation in treated cells (Figure 2I–L), and the proportion of damaged cells increased with exposure time (Figure 2M). To evaluate the effect on key cellular components, bacterial protein and DNA were exposed to CAP. While CAP had no significant impact on DNA integrity or concentration (Figure 2N,O), it caused time-dependent protein degradation (Figure 2P,Q). Collectively, these results illustrate that CAP preferentially damages bacterial internal structures rather than external components.

### 3.3. CAP Disinfects S. aureus via a Reactive Species-Dependent Mechanism

**Figure 3:**
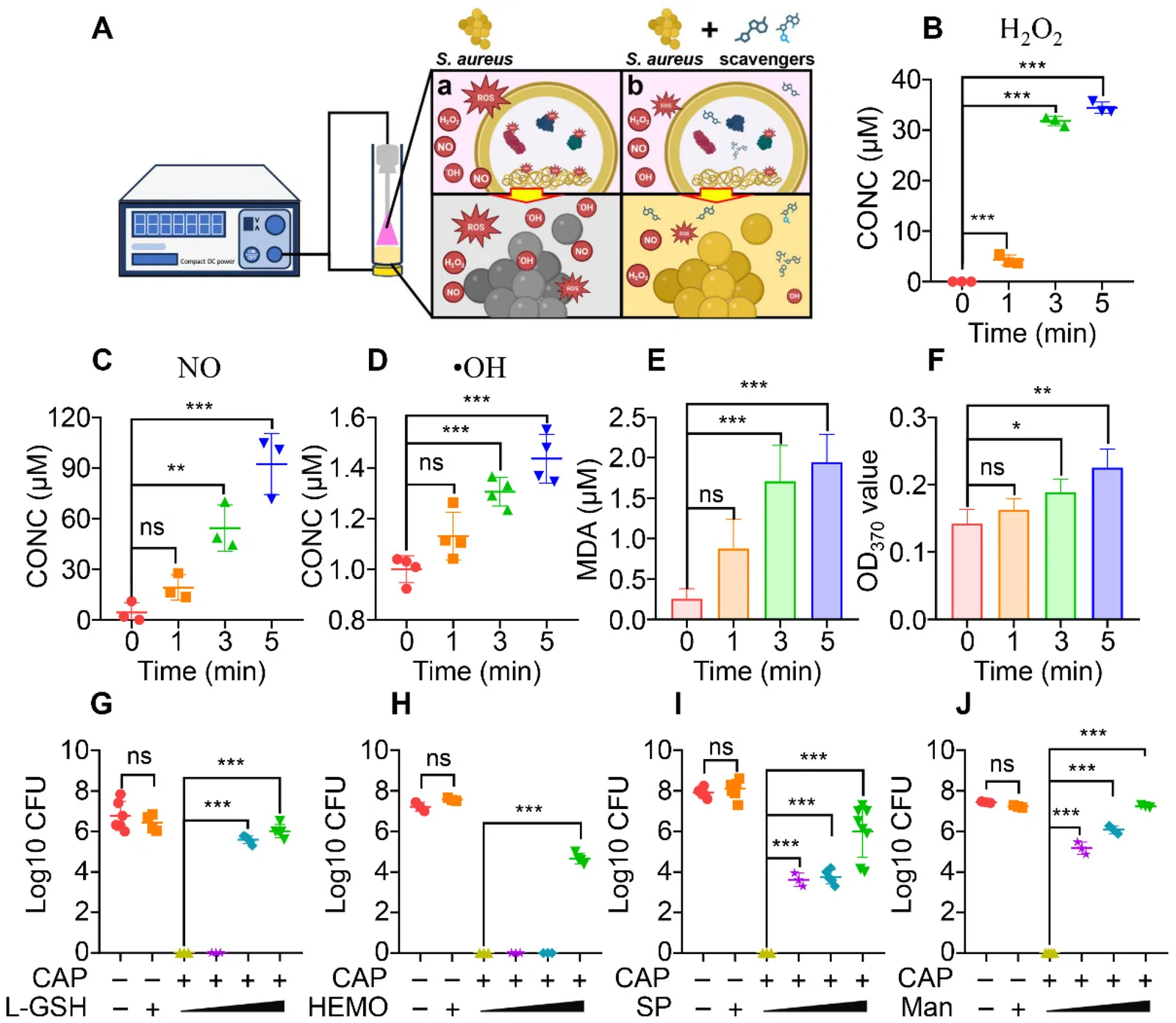
CAP disinfects *S. aureus* through reactive species-triggered oxidative damage. (**A**) Schematic showing that CAP-derived reactive species trigger oxidative damage (**a**), which can be inhibited by exogenous ROS scavengers (**b**). (**B**–**D**) Quantification of (**B**) hydrogen peroxide (H_2_O_2_), (**C**) nitric oxide (NO), and (**D**) hydroxyl radical (•OH) in CAP-activated normal saline. (**E**) Lipid malondialdehyde (MDA) levels, a marker of lipid oxidation, in CAP-treated bacteria. (**F**) Protein carbonylation levels, a marker of protein oxidation, in CAP-treated bacteria. (**G**–**J**) Effect of ROS scavengers on CAP bactericidal activity: (**G**) reduced L-glutathione (L-GSH, 0, 1, 10, and 100 mM), (**H**) hemoglobin (HEMO, 0, 0.01, 0.1, 1 mM), (**I**) sodium pyruvate (SP, 0, 1, 10, 100 mM), and (**J**) mannitol (Man, 0, 0.01, 0.1, 1 mM). Data are presented as mean ± SD. Statistical significance was calculated by one-way ANOVA. “ns” indicates no significance, * *p* < 0.05, ** *p* < 0.01, *** *p* < 0.001.

CAP generates diverse free radicals, including long-lived (e.g., H_2_O_2_, NO_2_^−^) and short-lived (e.g., •OH, •NO) reactive species, which can induce oxidative damages (Figure 3A) [10]. Quantitative analysis of representative reactive species (H_2_O_2_, NO, and •OH) in CAP-treated systems showed significant time-dependent increases: after 5 min of CAP exposure, the concentrations of H_2_O_2_, NO, and •OH were 34.4-fold, 14.2-fold, and 1.4-fold higher than initial levels, respectively (Figure 3B–D). Then, we detected the CAP-induced oxidative damages to bacterial components. The levels of lipid malondialdehyde (MDA) and protein carbonylation, key markers of oxidative damage, were dramatically elevated with prolonged CAP exposure, reaching 7.6-fold and 1.6-fold of control levels after 5 min of CAP treatment, respectively (Figure 3E,F). These data suggest that CAP disinfects *S. aureus* via reactive species-dependent oxidative stress.

To confirm the role of reactive species, we tested the effect of ROS scavengers (L-GSH, HEMO, SP, and Man) on CAP bactericidal activity (Figure 3A). Individual scavengers had a minimal effect on bacterial growth (Figure 3G–J). In contrast, each of these four agents, when combined with CAP, markedly reduced the disinfection effect of CAP. Notably, L-GSH (>10 mM), SP (>1 mM), and Man (>0.01 mM) exhibited does-dependent inhibition of CAP efficacy, whereas HEMO (<0.1 mM) had no effect. Together, these data further validate the oxidative mechanism underlying CAP-mediated *S. aureus* disinfection.

### 3.4. Disruption of Antioxidant Genes Enhances S. aureus Susceptibility to CAP

**Figure 4:**
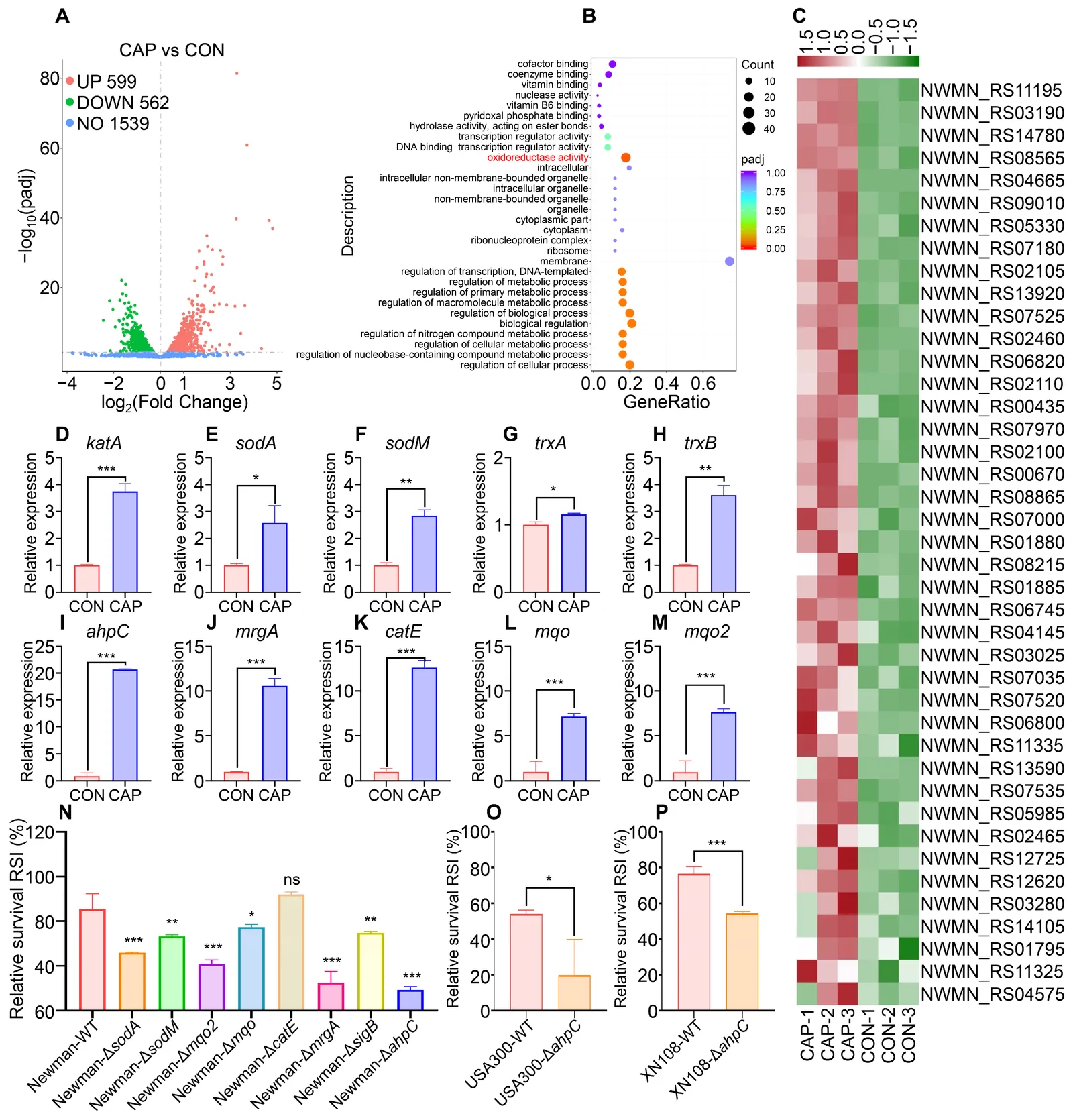
Disruption of antioxidant genes enhances *S. aureus* susceptibility to CAP. (**A**) Volcano plot of gene expression changes in *S. aureus* Newman after 5 min CAP treatment (RNA-seq data). (**B**) Gene Ontology (GO) enrichment analysis of upregulated genes. (**C**) Heatmap of expression levels of oxidoreductase activity-related genes upregulated in CAP-treated (CAP) vs. control (CON) groups. (**D**–**M**) RT-qPCR validation of CAP-induced upregulation of key antioxidant genes. (**N**) Bactericidal effect of 90 s CAP on *S. aureus* antioxidant-gene deletion mutants. (**O**,**P**) *ahpC* deletion enhances CAP susceptibility in *S. aureus* strains USA300 (**O**) and XN108 (**P**). Relative survival index (RSI) = log_10_(survival count after CAP)/log_10_(initial count before CAP). Data are presented as mean ± SD. Statistical significance was calculated by unpaired Student’s *t*-test or one-way ANOVA. “ns” indicates no significance, * *p* < 0.05, ** *p* < 0.01, *** *p* < 0.001.

To investigate bacterial responses to CAP-induced oxidative stress, RNA-seq was performed on *S. aureus* Newman before and after 5 min of CAP treatment. This treatment altered the expression of 1161 genes (599 upregulated, 562 downregulated; Figure 4A). Gene Ontology (GO) enrichment and gene set enrichment analysis (GSEA) consistently demonstrated significant enrichment of upregulated genes associated with oxidoreductase activity (Figure 4B and Figure S2), with the expression of 41 redox-related genes significantly elevated (Figure 4C). RT-qPCR validated the upregulation of key antioxidant genes, with fold changes ranging from 1.16 (*trxA*) to 20.68 (*ahpC*) relative to untreated controls (Figure 4D–M). These findings indicate that *S. aureus* activates its antioxidant system in response to CAP-induced oxidative stress.

To elucidate the role of these upregulated antioxidants, we constructed deletion mutants (Newman-Δ*sodA*, Δ*sodM*, Δ*mqo2*, Δ*mqo*, Δ*catE*, Δ*mrgA*, Δ*sigB*, and Δ*ahpC*) via homologous recombination (Figure S3). After 90 s CAP exposure, all mutants (except Newman-Δ*catE*) exhibited reduced viability compared to the wild-type control (Figure 4N and Figure S4), with Newman-Δ*ahpC* exhibiting the lowest survival rate (49.3%). Notably, *ahpC* deletion in two additional *S. aureus* strains (XN108 and USA300) also increased CAP susceptibility (Figure 4O,P). These results suggest that inactivation of antioxidant factors, particularly AhpC, effectively enhances CAP-mediated bacterial eradication.

### 3.5. AhpC Inhibitor C4515 Potentiates CAP Disinfection of S. aureus

**Figure 5:**
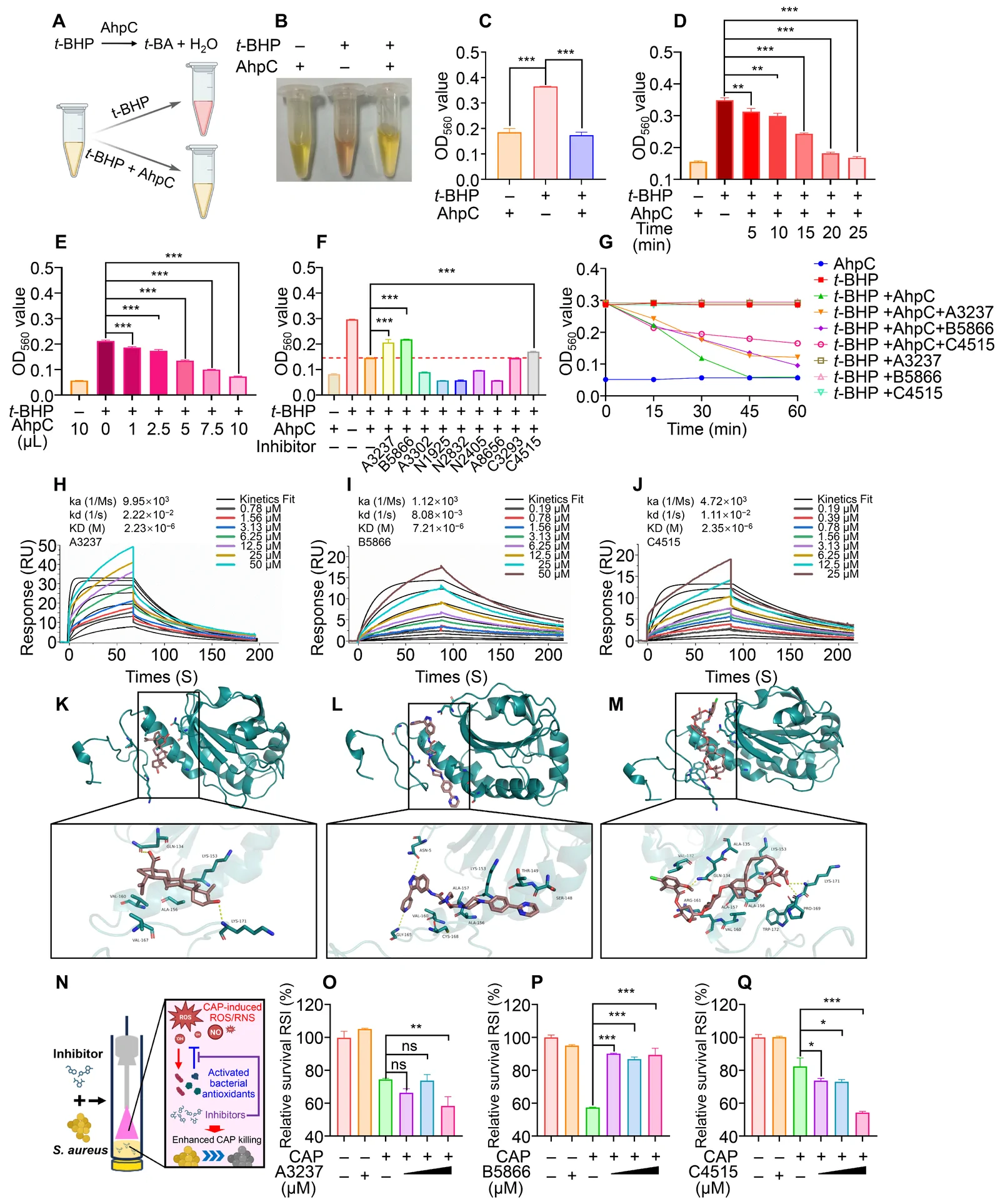
AhpC inhibitors potentiate CAP disinfection of *S. aureus*. (**A**) Schematic of AhpC-mediated degradation of *t*-butyl hydroperoxide (*t*-BHP) and the resulting color change. (**B,C**) In vitro detection of AhpC peroxidase activity: (**B**) representative images of reaction tubes 30 min post-incubation. (**C**) OD_560_ of reaction systems 30 min post-incubation. (**D**) Time-course OD_560_ (measured every 5 min) of systems containing 10 μL of AhpC. Statistical comparisons are made versus the positive control (*t*-BHP only). (**E**) OD_560_ 30 min post-incubation with varying amounts of AhpC. (**F**) OD_560_ 30 min post-incubation with candidate small-molecule inhibitors. (**G**) Time-course OD_560_ (every 15 min) of systems containing A3237, B5866, or C4515. (**H**–**J**) Multi-concentration SPR analysis of binding affinities between AhpC and (**H**) A3237, (**I**) B5866, and (**J**) C4515 (small molecule concentrations are indicated). (**K**–**M**) Molecular docking analysis of AhpC interactions with (**K**) A3237, (**L**) B5866, and (**M**) C4515. (**N**) Schematic showing the mechanism of enhanced CAP bactericidal effect. CAP-induced ROS upregulate bacterial antioxidants that in turn scavenge ROS. The small-molecule inhibitor suppresses these factors, blocking ROS scavenging and enhancing CAP-mediated killing. (**O**–**Q**) Potentiation of CAP-mediated killing of *S. aureus* by (**O**) A3237, (**P**) B5866, and (**Q**) C4515. Relative survival index (RSI) = log_10_(survival count after CAP)/log_10_(initial count before CAP). Data are presented as mean ± SD. Statistical significance was calculated by one-way ANOVA. “ns” indicates no significance, * *p* < 0.05, ** *p* < 0.01, *** *p* < 0.001.

AhpC is a key antioxidant in *S. aureus* that detoxifies peroxides and H_2_O_2_ [28]. Recombinant AhpC was prepared (Figure S5) and shown to effectively degraded *t*-butyl hydroperoxide (*t*-BHP) in vitro, as reflected by color changes and reduced OD_560_ (optical density at 560 nm) values (Figure 5A–C). AhpC catalytic activity was positively correlated with reaction duration and protein concentration (Figure 5D,E and Figure S6A,B), confirming the robust enzymatic activity of the purified AhpC protein.

Virtual screening of the L-CO-020 small-molecule library (~10,000 compounds; Supplementary Data S1) was performed to identify AhpC inhibitors. Ten candidates were selected based on biological function, solubility, and molecular weight (Table S3). The inhibitory effects of these candidates, excluding A3832 which caused turbidity, were evaluated, and three compounds (A3237, B5866, and C4515) significantly inhibited AhpC-mediated *t*-BHP degradation in a time-dependent manner (Figure 5F,G). Multi-concentration SPR analysis confirmed dose-dependent binding of these small-molecules to AhpC, with dissociation constants (Kd) of 2.23 × 10^−6^ (A3237), 7.21 × 10^−6^ (B5866), and 2.35 × 10^−6^ (C4515) (Figure 5H–J). Molecular docking revealed the binding of these molecules to the active site of AhpC (Figure 5K–M), validating them as promising AhpC inhibitors.

When tested for their ability to enhance CAP disinfection efficacy (Figure 5N), individual application of A3237, B5866, or C4515 had no impact on bacterial growth (Figure 5O–Q and Figure S7). However, co-treatment of each compound with CAP yielded quite distinct outcomes: A3237 (500 μM) and C4515 (all tested concentrations) significantly enhanced the bactericidal effect of CAP, with C4515 reducing bacterial survival from 82% to 54%. In contrast, B5866 failed to enhance CAP activity and even protected bacteria, with mechanisms to be elucidated. These results identify C4515 as a potent AhpC inhibitor that sensitizes *S. aureus* to CAP disinfection.

### 3.6. MrgA Inhibitor A8226 Promotes CAP Disinfection of S. aureus

**Figure 6:**
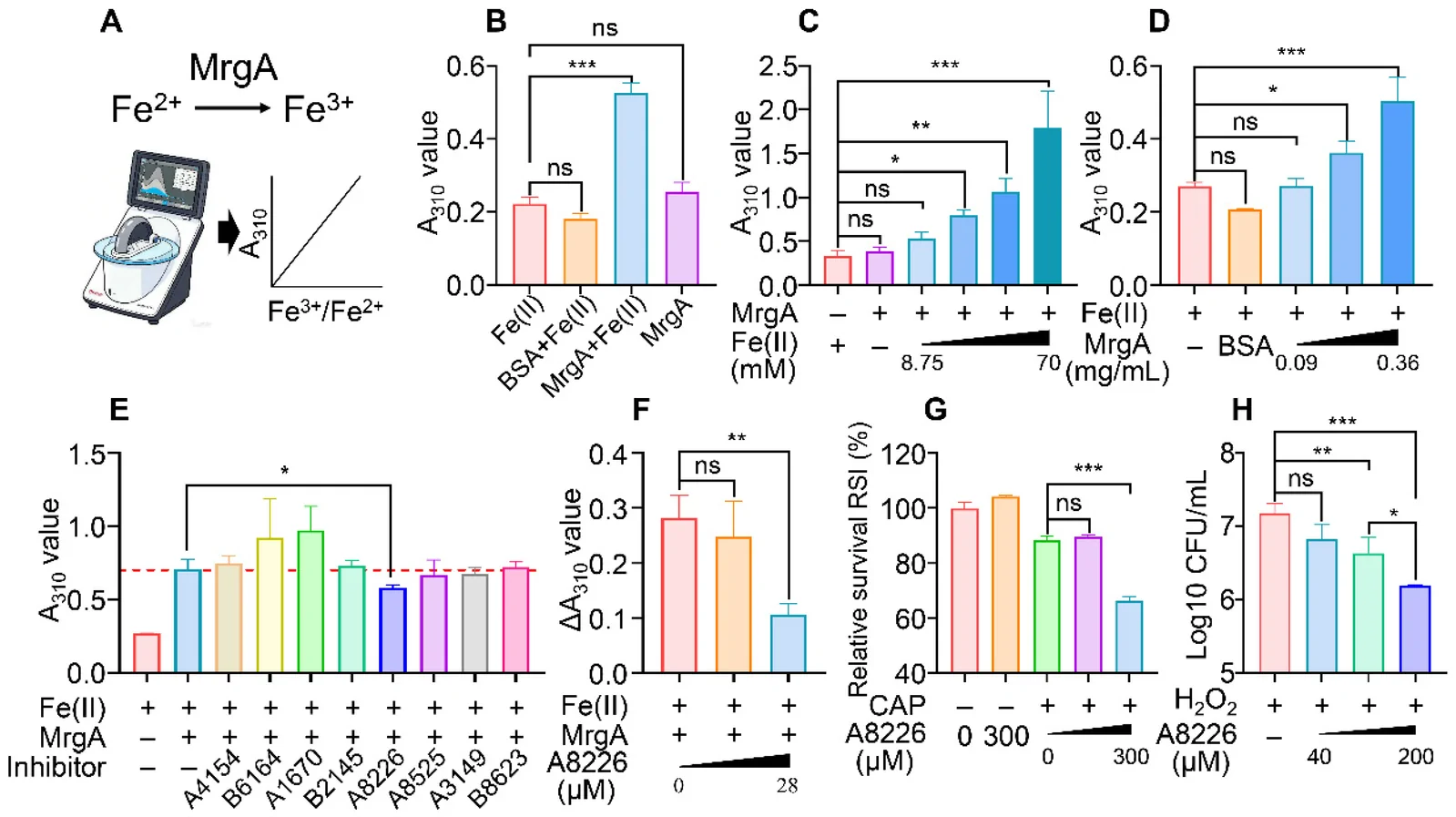
MrgA inhibitors potentiate CAP disinfection of *S. aureus*. (**A**) Schematic of MrgA ferroxidase activity assay. MrgA catalyzes Fe^2+^ → Fe^3+^ conversion, resulting in increased A_310_ value. (**B**) In vitro detection of MrgA ferroxidase activity (BSA as a negative control); A_310_ was measured immediately after reaction. (**C**) A_310_ measured immediately after reaction with varying Fe^2+^ amounts and fixed MrgA. (**D**) A_310_ measured immediately after reaction with varying MrgA concentrations and fixed Fe^2+^. (**E**) A_310_ measured immediately after reaction with candidate small molecules. Statistical comparisons are made versus the positive control (Fe^2+^ + MrgA). (**F**) Dose-dependent effect of A8226 on MrgA ferroxidase activity (ΔA_310_ = A_310_ of test group − A_310_ of negative control). (**G**) Potentiation of CAP-mediated killing by A8226: *S. aureus* Newman (1 × 10^8^ CFU/mL) was pre-incubated with A8226 for 30 min, followed by 90 s CAP treatment. Relative survival index (RSI) = log_10_(survival count after CAP)/log_10_(initial count before CAP). (**H**) Potentiation of H_2_O_2_-mediated killing by A8226: *S. aureus* Newman (1 × 10^8^ CFU/mL) was pre-incubated with A8226 for 30 min, followed by 3% H_2_O_2_ treatment for 30 min; survival was quantified via plate dilution. Data are presented as mean ± SD. Statistical significance was calculated by one-way ANOVA. “ns” indicates no significance, * *p* < 0.05, ** *p* < 0.01, *** *p* < 0.001.

The Newman-Δ*mrgA* also exhibited increased CAP sensitivity (Figure 4N). MrgA prevents hydroxyl radical formation during the Fenton reaction [29]. Recombinant MrgA was purified (Figure S8), and its ferroxidase activity was confirmed by catalyzing the Fe^2+^ → Fe^3+^ conversion, evidenced by an increase in absorbance at 310 nm (A_310_) (30), a function absent in control BSA (Figure 6A,B). MrgA ferroxidase activity was positively correlated with F^2+^ and protein concentrations, with a significant A_310_ elevation observed at Fe^2+^ concentrations > 17.5 mM and MrgA concentrations > 0.18 mg/mL (Figure 6C,D).

Virtual screening of the L-CO-020 library (Supplementary Data S2) identified eight candidate MrgA inhibitors (Table S4), all of which docked to MrgA’s active region (Figure S9). However, only A8226 slightly reduced MrgA ferroxidase activity in a dose-dependent manner (Figure 6E,F). Individual A8226 treatment did not affect bacterial replication (Figure 6G), but co-treatment with either CAP or H_2_O_2_ significantly enhanced bactericidal efficacy against *S. aureus* (Figure 6G,H and Figure S10). Collectively, these results support the combination of MrgA antagonists and CAP as an effective disinfection strategy for *S. aureus*.

## 4. Discussion

As target-dependent antibacterial agents, antibiotics are gradually losing their efficacy against bacterial infections, primarily due to pathogen mutations altering drug target sites. Once the drug targets mutate, bacterial resistance occurs [30,31]. In contrast, CAP eradicates bacteria via diverse reactive species, particularly ROS and RNS, which exert broad oxidative damage [15]. This non-specific mode of action likely underlies the reported inability of bacteria to develop resistance following repeated CAP exposure [32,33], highlighting CAP as a promising alternative for combating AMR infections. However, bacteria exhibit intrinsic CAP tolerance. We observed clear heterogeneity in CAP sensitivity among *S. aureus* strains, consistent with reports across bacterial species [27,34,35]. Therefore, we sought to identify key factors mediating CAP tolerance in *S. aureus*.

Previous investigations have explored mechanisms of bacterial defense against CAP. Several studies have proposed morphology-based mechanisms, as Gram-positive bacteria generally exhibit higher CAP tolerance than Gram-negative bacteria [36]. The thicker peptidoglycan layer in Gram-positive bacteria may offer protection against direct attack and electrostatic disruption from charged particles and reactive species [37,38,39]. In our study, however, despite notable variations in bacterial survival, SEM analysis revealed no distinct CAP-induced changes in surface structures, indicating that factors beyond cell wall integrity primarily govern CAP resistance in *S. aureus*. Other studies report that *S. aureus* strains carrying the *mecA* gene exhibit greater resistance to CAP-induced killing than their isogenic counterparts [35]. Given that *mecA* is closely associated with β-lactam resistance in MRSA, and our data show comparable CAP efficacy against MRSA and MSSA, we conclude that antibiotic resistance-related genes do not confer cross-resistance to CAP. Instead, we propose that the expression pattern of antioxidant genes determines this resistance phenotype, based on three key lines of evidence. First, CAP primarily kills Gram-positive bacteria by inducing oxidative damage. We quantified reactive species in CAP-activated solutions and within cells, confirming oxidative damage to proteins and lipids. Second, RNA-seq and RT-qPCR analyses demonstrated significant upregulation of oxidoreductase-related genes. In line with these findings, several studies have reported that CAP exposure promotes the expression of antioxidant genes such as *katA* and *trxA* [40,41], indicating bacterial activation of antioxidant defenses. Third, and most importantly, the CAP–bacteria interaction is essentially governed by the antagonism between CAP-derived reactive species and staphylococcal endogenous antioxidant systems. Disrupting this balance either by adding ROS scavengers or by deleting antioxidant genes significantly alters CAP treatment outcome. Thus, increasing bacterial sensitivity to CAP is crucial for improving its sterilization efficacy.

Inspired by the clinical success of pairing β-lactams with β-lactamase inhibitors to combat antibiotic resistance [42], we developed a novel disinfection strategy that combines CAP with small-molecule inhibitors targeting *S. aureus* antioxidant factors, both approaches rely on increasing bacterial sensitivity to antimicrobials. AhpC was identified as a core antioxidant component in this study. Further screening revealed that C4515, a candidate AhpC inhibitor, effectively improved CAP disinfection without elevating CAP intensity. AhpC functions as a peroxidase with a homodimer serving as its minimal functional unit; within this dimer, the C46–C165 (C49–C168 in *S. aureus*) intersubunit disulfide bond acts as a key intermediate required for sustained H_2_O_2_-decomposing activity [28]. Molecular docking predicts that C4515 binds to the C-terminal tail of AhpC, a region in close spatial proximity to residue C168. Ligand binding at this site may hinder formation of the C49–C168 disulfide bond, thereby reducing AhpC activity and rendering bacteria more susceptible to CAP-mediated killing. For the other key antioxidant protein MrgA, the candidate inhibitor A8226 elicits a comparable sensitizing effect. Since MrgA functions as a dodecamer [25], predicted binding of A8226 to the exposed N-terminal α-helix of the MrgA monomer may disrupt dodecamer assembly and attenuate its ferroxidase activity. These results demonstrate that this “double attack” strategy is feasible via targeting distinct antioxidant defense factors, significantly expanding molecular options for CAP sensitization of *S. aureus* and potentially extending to other drug-resistant Gram-positive bacteria. In contrast, Gram-negative pathogens exhibit intrinsic susceptibility to CAP and may not require this type of sensitization regimen. Additionally, numerous studies have reported that combining CAP with various agents, such as antibiotics, bacteriocins, and plant extracts [43,44,45], enhances its disinfection efficacy. Collectively, these findings demonstrate that CAP-based combination therapy represents a potent and versatile antibacterial strategy worthy of further investigation.

Nevertheless, our study has several limitations, including unresolved issues and observations that differ from some previous reports. While previous studies have revealed that CAP treatment drives bacteria into a viable but non-culturable (VBNC) state [40,46], we observed that CAP-treated bacteria recovered to control-level growth, a discrepancy possibly attributable to the relatively low CAP intensity applied in our experiment. This further emphasizes the importance of increasing bacterial sensitivity to improve CAP sterilization efficiency. Furthermore, although this combination strategy has been validated in vitro, the in vivo effectiveness remains unconfirmed, partly because the probe-electrode CAP device cannot be directly applicable to animal models. Future studies should adopt an appropriate jet-type CAP device to verify the in vivo efficacy and safety of this combination strategy. In addition, our evaluation of CAP bactericidal activity was limited to planktonic *S. aureus* in saline. Further investigations are required to examine treatment performance under more physiologically relevant conditions, including bacteria attached to solid surfaces, exposed to organic contaminants, or embedded within biofilms, where penetration of CAP-derived reactive species can be substantially impaired [47].

## 5. Conclusions

In conclusion, this study confirms that CAP exerts potent bactericidal activity against both antibiotic-sensitive and resistant *S. aureus* via ROS-dependent oxidative damage. Upon CAP challenge, *S. aureus* activates antioxidant defense systems, prominently including AhpC and MrgA, to counteract oxidative stress. We identified two small-molecule inhibitors, C4515 and A8226, which target AhpC and MrgA, respectively. Co-treatment with C4515 markedly potentiates CAP disinfection efficacy and reduces bacterial survival from 82% to 54%, demonstrating that combining redox-targeting small molecules with CAP represents a promising therapeutic strategy against recalcitrant staphylococcal pathogens. These findings deepen our molecular understanding of plasma-bacterium interactions and deliver proof-of-concept evidence for combinatorial approaches to combat clinically relevant drug-resistant bacterial infections, laying a foundation for future translational investigation.

## Data Availability

The original data presented in the study are openly available in the NCBI SRA database under the accession number PRJNA1503662 (https://www.ncbi.nlm.nih.gov/sra/PRJNA1503662 (accessed on 29 July 2026)).

## Supplementary Materials

The following supporting information can be downloaded at: https://www.mdpi.com/article/10.3390/antiox15101316/s1, Table S1: Information of the S. aureus strains used in this study. Table S2: Information of primers used in this study. Table S3: Information of selected 10 candidate small-molecular inhibitors of AhpC. Table S4: Information of selected 8 candidate small-molecular inhibitors of MrgA. Figure S1: The growth curve of CAP-treated S. aureus Newman. Figure S2: CAP treatment upregulated expression of genes associated with oxidoreductase activity. Figure S3: Construction and identification of antioxidant gene mutants based on S. aureus Newman. Figure S4: CAP exposure of S. aureus Newman and its antioxidant-gene deletion mutants. Figure S5: SDS-PAGE analysis of purified recombinant S. aureus MrgA protein. Figure S6: Detection of the peroxidase activity of AhpC protein in vitro. Figure S7: AhpC inhibitor potentiates CAP-mediated disinfection of S. aureus. Figure S8: SDS-PAGE analysis of purified recombinant S. aureus MrgA protein. Figure S9: Molecular docking analysis of MrgA-candidate inhibitor interaction. Figure S10: MrgA inhibitor attenuates the antioxidant capacity of S. aureus. Data S1. Predicted binding affinities of compounds from the L-CO-020 library against AhpC, ranked by docking affinity (kcal/mol). Data S2. Predicted binding affinities of compounds from the L-CO-020 library against MrgA, ranked by docking affinity (kcal/mol). Refs. [48,49,50,51] are cited in Supplementary Materials File.
