# Supplementary material for: Small-Molecule Inhibitors Targeting Bacterial Antioxidants Potentiate Bactericidal Efficacy of Cold Atmospheric Plasma Against Staphylococcus aureus
Source: Antioxidants (Basel). 2026 Oct 12;15(10):1316. doi: 10.3390/antiox15101316 (PMC13701694; doi:10.3390/antiox15101316)
Supplement: Supplementary file 1 [file antioxidants-15-01316-s001.zip › 3-Supplementary Material-Antioxidants-revised.pdf]

Table S1. Information of the *S. aureus* strains used in this study

Strains	Comments	Reference
Newman	A strain isolated from a human infection and has been used extensively in animal models of staphylococcal disease.	[26]
RN4220	A laboratory strain with defects in type I restriction-modification system and is used for performing genetic operations. A gift from Prof. Baolin Sun (University of Science and Technology of China).	[48]
USA300	A clone of meticillin-resistant *Staphylococcus aureus* and is a major source of community-acquired infections in the USA, Canada, and Europe.	[49]
XN108	The first ST239 VISA strain isolated in mainland China. Kept in our laboratory. Vancomycin MIC=12 mg/L.	[50]
Newman-Δ*sodA*	Deletion the *sodA* gene in Newman	This study
Newman-Δ*sodM*	Deletion the *sodM* gene in Newman	This study
Newman-Δ*mqo*	Deletion the *mqo* gene in Newman	This study
Newman-Δ*mqo2*	Deletion the *mqo2* gene in Newman	This study
Newman-Δ*catE*	Deletion the *catE* gene in Newman	This study
Newman-Δ*mrgA*	Deletion the *mrgA* gene in Newman	This study
Newman-Δs*igB*	Deletion the s*igB* gene in Newman	[51]
Newman-Δ*ahpC*	Deletion the *ahpC* gene in Newman	This study
XN108-Δ*ahpC*	Deletion the *ahpC* gene in XN108	This study
USA300-Δ*ahpC*	Deletion the *ahpC* gene in USA300	This study

Table S2. Information of primers used in this study

Primer	Sequence	Description
katA-1	5'-GTCTTCCAGAAGCATTGCACCA-3'	RT-qPCR detection of
katA-2	5'-GCGTTGAGATGAATCACGATCTG-3'	*katA* expression
sodA-1	5'-GACAGTGTACCAGCTAACATCCAAAC-3'	RT-qPCR detection of
sodA-2	5'-GGTAATAAGCGTGTTCCCATACGT-3'	*sodA* expression
sodM-1	5'-GCGGATATGATTGCTAACTTAGACAA-3'	RT-qPCR detection of
sodM-2	5'-CATCTAAAGTGCCCCACTGCG-3'	*sodM* expression
trxA-1	5'-GTAGAATCTGGTGTACAACTAGTAGA-3'	RT-qPCR detection of
trxA-2	5'-ACTTCATATTTAGCTGCAGTTGATGG-3'	*trxA* expression
trxB-1	5'-GAAGTAGAGAACTTCCCTGGTTTC-3'	RT-qPCR detection of
trxB-2	5'-CTGCACCTGTAGCAATAATAACCG-3'	*trxB* expression
ahpC-1	5'-GAGCGTAGTATGCTTCTATCCTG-3'	RT-qPCR detection of
ahpC-2	5'-GTGATTGTTTGTGATGGGTCACC-3'	*ahpC* expression
mrgA-1	5'-TACACGTTAAATTTGAAGAA-3'	RT-qPCR detection of
mrgA-2	5'-CTTCAACCATTTGTTCTGCA-3'	*mrgA* expression
catE-1	5'-CGTGAACCTTTAATGTCCGA-3'	RT-qPCR detection of
catE-2	5'-GTCCATTGCGCTTCGTCT-3'	*catE* expression
mqo-1	5'-GCACAACCTGGCGAAGAG-3'	RT-qPCR detection of
mqo-2	5'-CACCGCTTGAATAAAGCGA-3'	*mqo* expression
mqo2-1	5'-AGGCCGTGAAGATAACCCTGG-3'	RT-qPCR detection of
mqo2-2	5'-CAGCACCGATGAATACGTAGTC-3'	*katA* expression
sodA-ko-1-1	5'-CGCGAATTCGTTGTAGGGATACTGTTAGT-3'	*sodA* gene upstream
sodA-ko-1-2	5'-CGCGGTACCAAATAATCATCCTCCTAA-3'	amplification
sodA-ko-2-1	5'-CGCGGATCCAGTATGATTACTTGTTCG-3'	*sodA* gene downstream
sodA-ko-2-2	5'-GCGTCGACCGATACATCTCCGAATATAC-3'	amplification

Primer	Sequence	Description
sodA-out-1	5’-GTAGCGATGGTTATTTCTGCCTCAA-3’	Detection of *sodA* gene
sodA-out-2	5’-CTTTTGGAATACCTTCAGCAGGTGT-3’	deletion
sodM-ko-1-1	5’-CGCGAATTCAATCACATAGTTTAACTAAA-3’	*sodM* gene upstream
sodM-ko-1-2	5’-CGCGGTACCAAGTATATTCCTCCTTTTAT-3’	amplification
sodM-ko-2-1	5’-CGCGGATCCTATAACTTAATATAATTGAG-3’	*sodM* gene downstream
sodM-ko-2-2	5’-GCGTCGACATCATTTGATGGTTTGATGA-3’	amplification
sodM-out-1	5’-CATACCAACAAGTTGGTATC-3’	Detection of *sodM* gene
sodM-out-2	5’-CTAGGACATTGATTTATGTCC-3’	deletion
mqo-ko-1-1	5’-GCCACTCATCGCAGTGCAGCACTTACCTGGAACAATCAGC-3’	*mqo* gene upstream
mqo-ko-1-2	5’-AGACATTACCGTTTCGTTTAATTAATACCACTTTAAATCA-3’	amplification
mqo-ko-2-1	5’-TGATTTAAAGTGGTATTAATTAAACGAAACGGTAATGTCT-3’	*mqo* gene downstream
mqo-ko-2-2	5’-GGTGCCTGACTGCGTTAGCAAACCTATGATGAAACTCAAT-3’	amplification
mqo-out-1	5’-CAGTCATTGTAATGATTTGGAGTG-3’	Detection of *mqo* gene
mqo-out-2	5’-ATATATCGATGGTCATTTATGGTT-3’	deletion
mqo2-ko-1-1	5’-CGCGAATTCGTTGACGACTTACCAAAAAC-3’	*mqo2*gene upstream
mqo2-ko-1-2	5’-CGCGGTACCTGGTTTCACCTCTCCAA-3’	amplification
mqo2-ko-2-1	5’-CGCGGATCCATTCTAATAGCTATATTTA-3’	*mqo2*gene downstream
mqo2-ko-2-2	5’-GCGTCGACAAACAATGGATCGCGAAG-3’	amplification
mqo2-out-1	5’-TGAAGTTGCACCATATAAATATCCGA-3’	Detection of *mqo2*gene
mqo2-out-2	5’-TTCACCTTCATTCTCACCAGATAAAA-3’	deletion
catE-ko-1-1	5’-GCCACTCATCGCAGTGCAGCACAACGTGATTCTCATATCC-3’	*catE* gene upstream
catE-ko-1-2	5’-TCGTCCATTCAAAGTATATATCAATTAACTCCTATCAAAT-3’	amplification
catE-ko-2-1	5’-ATTTGATAGGAGTTAATTGATATATACTTTGAATGGACGA-3’	*catE* gene downstream
catE-ko-2-2	5’-GGTGCCTGACTGCGTTAGCACACTTCAAATGAATCTCCAA-3’	amplification
catE-out-1	5’-TTAGGTAATGTGATTGTACCTGACC-3’	Detection of *catE* gene

Primer	Sequence	Description
catE-out-2	5’-CATGCAGCACCATTATTTCG-3’	deletion
mrgA-ko-1-1	5’-AGCTCGGTACCCGGGGATCCCCAGTGGTATGTTGAATAAG-3’	*mrgA* gene upstream
mrgA-ko-1-2	5’-ATATTATTACTTATCTGTAGAATTTAATACACTCCTTAAAAT-3’	amplification
mrgA-ko-2-1	5’-ATTTTAAGGAGTGTATTAAATTCTACAGATAAGTAATAATAT-3’	*mrgA* gene downstream
mrgA-ko-2-2	5’-CTTGCATGCCTGCAGGTCGACCAAAAGGTACACCACATATTC-3’	amplification
mrgA-out-1	5’-ACCATAAGAGCTAATTAAGAG-3’	Detection of *mrgA* gene
mrgA-out-2	5’-GACAACAAGCTAATCAGGATT-3’	deletion
ahpC-ko-1-1	5’-AGCTCGGTACCCGGGGATCCTGGAAGACATGGTCAAATGG-3’	*ahpC* gene upstream
ahpC-ko-1-2	5’-AGCATGGTTGTAATGCCTCCAAATATCTTCCTCCTAAGAA-3’	amplification
ahpC-ko-2-1	5’-TTCTTAGGAGGAAGATATTTGGAGGCATTACAACCATGCT-3’	*ahpC* gene downstream
ahpC-ko-2-2	5’-CTTGCATGCCTGCAGGTCGACCAATTGCTCTTCACCTGGAA-3’	amplification
ahpC-out-1	5’-GGTCGTAATCTGCCATTTGTG-3’	Detection of *ahpC* gene
ahpC-out-2	5’-AGAGGTCCGTCACAGTGAGG-3’	deletion
ahpC-cds-1	5’-TGCCGCGCGGCAGCCATATGATGTCATTAATTAACAAAGAAATCTTA-3’	*ahpC* cds amplification
ahpC-cds-2	5’-TGGTGGTGGTGGTGCTCGAGGATTTTACCTACTAAATCTAAACCAGG-3’	
mrgA-cds-1	5’-TGCCGCGCGGCAGCCATATGATGAGTAATCAACAAGATGTTGTAAA-3’	*mrgA* cds amplification
mrgA-cds-2	5’-TGGTGGTGGTGGTGCTCGAGGCTTAAGTAAGATTTAAACATCCAAT-3’	

Table S3. Information of selected 10 candidate small-molecular inhibitors of AhpC

No.	Name	Molecular weight	Affinity (kCal/mol)	Classification
C4515	Chlorothricin	955.5	-9.514	Microbiology & Virology|Antibiotic
N1925	Polyphyllin D	738.9	-9.095	Natural Products
A3302	CGI-1746	579.71	-8.9	Angiogenesis|BTK
N2832	Wilforlide A	454.68	-8.827	Natural Products
N2405	Ruscogenin	430.63	-8.684	Natural Products
A8656	Jervine	425.6	-8.68	Stem Cell|Hedgehog
A3237	Betulinic acid	456.7	-8.58	Apoptosis|Apoptosis Inducers
B5866	SCH772984 HCl	624.17	-8.568	MAPK Signaling|MEK1/2
C3293	Tomatidine	452.11	-8.539	–
A3832	Solasodine	413.64	-8.442	Others

Table S4. Information of selected 8 candidate small-molecular inhibitors of MrgA

No.	Name	Molecular weight	Affinity (kCal/mol)	Classification
A1670	Enzastaurin (LY317615)	515.61	-8.75	TGF-β / Smad Signaling|PKC
A4154	Olaparib (AZD2281, Ku-0059436)	434.46	-7.578	Chromatin/Epigenetics|PARP
B6164	A-1331852	658.81	-7.563	Apoptosis|Bcl-xL
B2145	Fosaprepitant dimeglumine salt	1004.83	-7.557	Neuroscience|Substance P/NK1 Receptor
A8226	TAK-632	554.52	-7.453	MAPK Signaling|Raf
A8525	Sotrastaurin (AEB071)	438.48	-7.444	TGF-β / Smad Signaling|PKC
B8623	Irinotecan hydrochloride	623.14	-7.448	–
A3149	AKT inhibitor VIII	551.64	-7.696	PI3K/Akt/mTOR Signaling|Akt

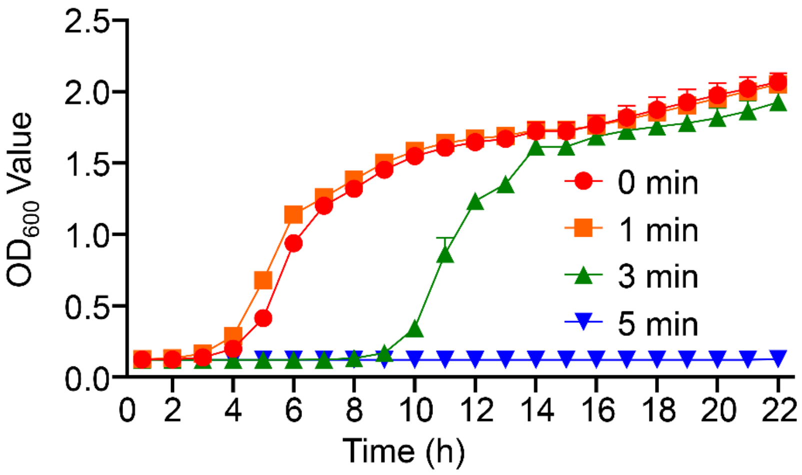

Figure S1. The growth curve of CAP-treated *S. aureus* Newman. Equal volumes of *S. aureus* Newman (approximately 1.0×10^8 CFU/mL) were subjected to CAP for different durations (0, 1, 3, 5 min), followed by a 1-h incubation at room temperature. Then, bacterial cells were transferred into fresh TSB medium and cultured at 37°C with shaking. The optical density at 600 nm (OD_{600}) was detected once every hour for 22 h. The growth curve of each sample was drawn. The experiment was repeated three times. Data were presented as mean ± SD.

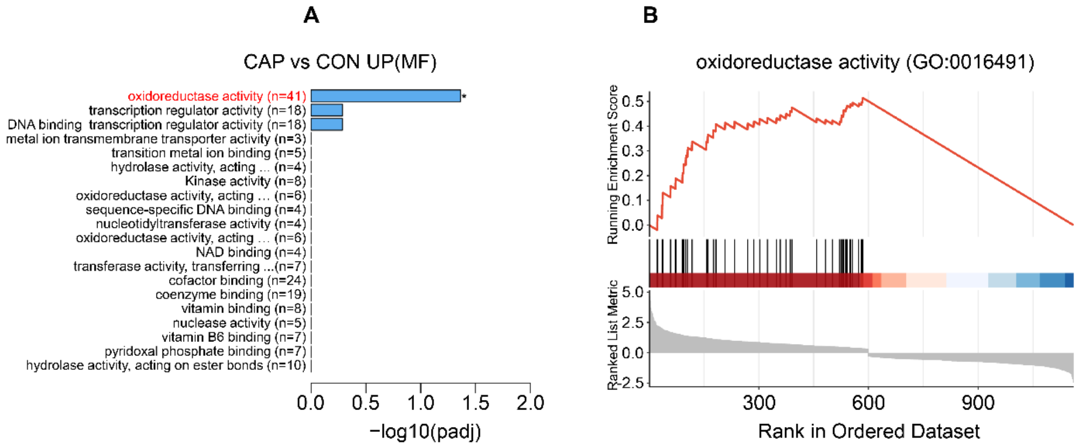

Figure S2. CAP treatment upregulated expression of genes associated with oxidoreductase activity. (A) Statistical analysis of gene sets enriched from MF-based GO analysis of genes upregulated by CAP treatment. (B) Gene set enrichment analysis (GSEA) of CAP-induced upregulated genes associated with oxidoreductase activity. The red line indicates enrichment profile.

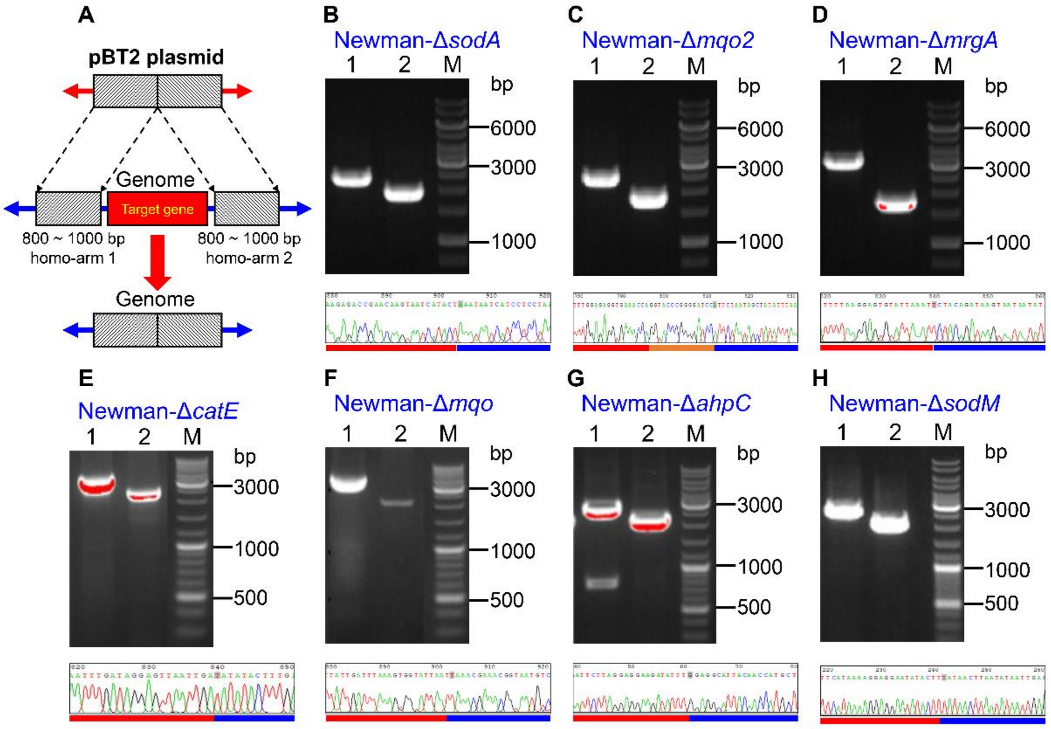

Figure S3. Construction and identification of antioxidant gene mutants based on *S. aureus* Newman. (A) Schematic diagram showing the strategy of homologous recombination for mutant strain construction. PCR confirming and DNA sequencing of the antioxidant gene mutants including (B) Newman-Δ*sodA*, (C) Newman-Δ*mqo2*, (D) Newman-Δ*mrgA*, (E) Newman-Δ*catE*, (F) Newman-Δ*mqo*, (G) Newman-Δ*ahpC*, and (H) Newman-Δ*sodM*. 1, wild strain; 2, mutants; M, DNA Marker. The red and blue bands under each sequencing profile indicated the upstream and downstream sequences flanking the target gene, respectively. The orange band corresponded to the sequence derived from the pBT2 plasmid.

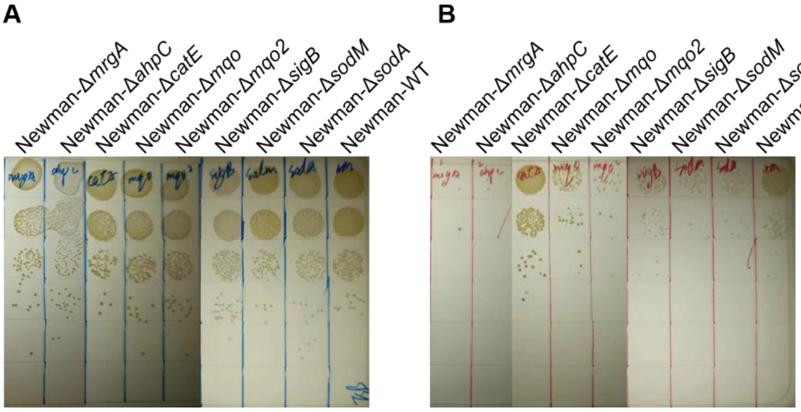

Figure S4. CAP exposure of *S. aureus* Newman and its antioxidant-gene deletion mutants. *S. aureus* Newman and its isogenic mutants were adjusted to approximately 1.0×10^8 CFU/mL. Viable bacterial counts (A) before and (B) after CAP exposure were counted via the plate dilution method.

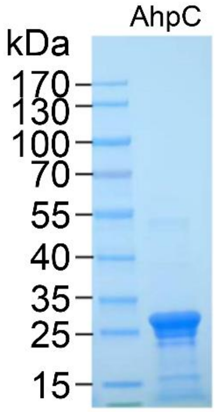

Figure S5. SDS-PAGE analysis of purified recombinant *S. aureus* MrgA protein.

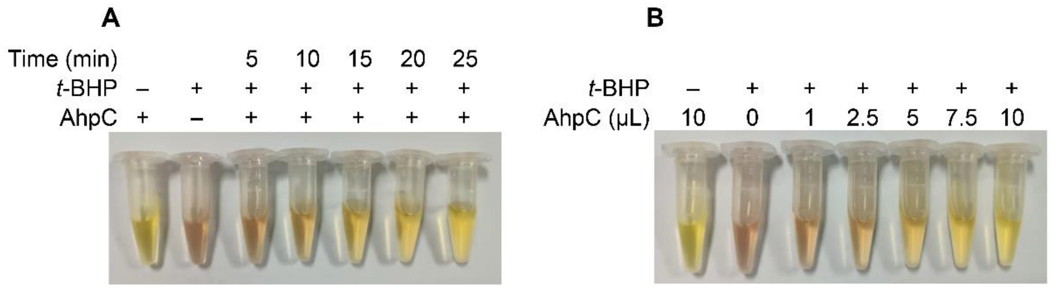

Figure S6. Detection of the peroxidase activity of AhpC protein in vitro. (A) Ten microliters of AhpC were added into reaction system, and the colors of the liquid in EP tubes with different reaction durations were photographed. (B) Varying amounts of AhpC were added into reaction system, and the colors of the liquid in different EP tubes were photographed 30 min after reaction. Tubes containing only AhpC or *t*-BHP served as negative and positive control, respectively. The experiments were repeated for three times.

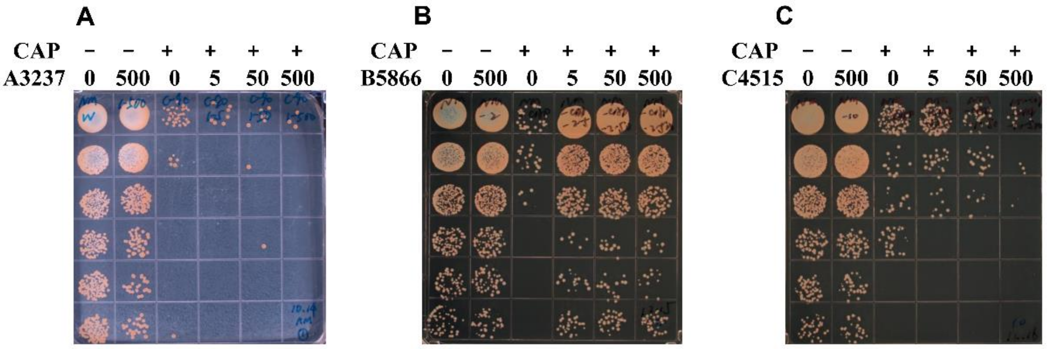

Figure S7. AhpC inhibitor potentiates CAP-mediated disinfection of *S. aureus*. *S. aureus* Newman was pre-incubated with (A) A3237, (B) B5866, and (C) C4515, followed by CAP treatment. Viable bacterial cells were counted via the plate dilution method.

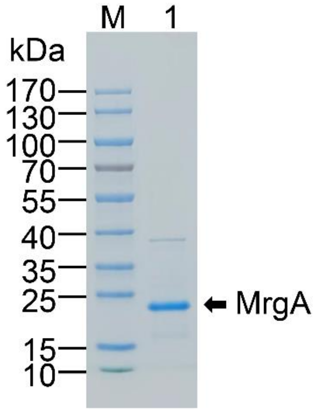

Figure S8. SDS-PAGE analysis of purified recombinant *S. aureus* MrgA protein.

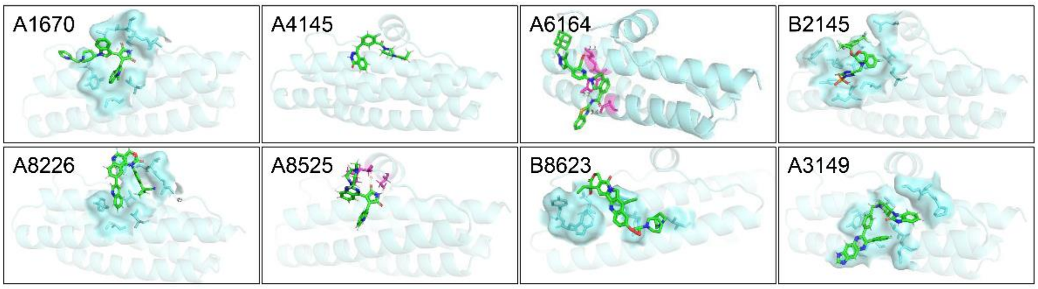

Figure S9. Molecular docking analysis of MrgA-candidate inhibitor interaction. The molecular interactions between MrgA protein and the eight candidate inhibitors were analyzed by using molecular docking technology. The number of each compound was marked in the box.

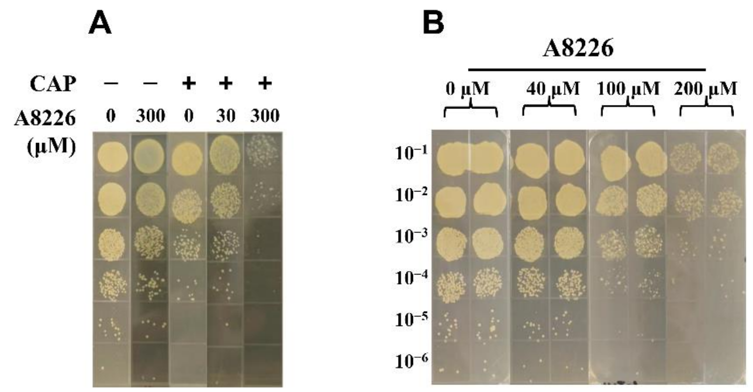

Figure S10. MrgA inhibitor attenuates the antioxidant capacity of *S. aureus*. *S. aureus* Newman (1×10^8 CFU/mL) was pre-incubated with the MrgA inhibitor A8266, followed by (A) CAP and (B) H_2O_2 treatment. Viable bacterial cells were counted via the plate dilution method.
